# Molecular features of premenopausal breast cancers in Latin American women: Pilot results from the PRECAMA study

**DOI:** 10.1371/journal.pone.0210372

**Published:** 2019-01-17

**Authors:** Magali Olivier, Liacine Bouaoun, Stephanie Villar, Alexis Robitaille, Vincent Cahais, Adriana Heguy, Graham Byrnes, Florence Le Calvez-Kelm, Gabriela Torres-Mejía, Isabel Alvarado-Cabrero, Fazlollah Shahram Imani-Razavi, Gloria Inés Sánchez, Roberto Jaramillo, Carolina Porras, Ana Cecilia Rodriguez, Maria Luisa Garmendia, José Luis Soto, Isabelle Romieu, Peggy Porter, Jamie Guenthoer, Sabina Rinaldi, on behalf of the PRECAMA team

**Affiliations:** 1 Section of Mechanisms of Carcinogenesis, International Agency for Research on Cancer, Lyon, France; 2 Section of Environment and Radiation, International Agency for Research on Cancer, Lyon, France; 3 Department of Pathology and Genome Technology Center, New York University Langone Medical Center, New York, United States of America; 4 Genetic Cancer Susceptibility Group, International Agency for Research on Cancer, Lyon, France; 5 Center for Population Health Research, National Institute of Public Health, Cuernavaca, Mexico; 6 Department of Pathology, Hospital de Oncología, Centro Médico Nacional Siglo XXI, Instituto Mexicano del Seguro Social, Mexico City, Mexico; 7 Department of Pathology, UMAE Hospital de Gineco Obstetricia No. 4 “Luis Castelazo Ayala”, Instituto Mexicano del Seguro Social, Mexico City, Mexico; 8 Group Infection and Cancer, School of Medicine, University of Antioquia, Medellín, Colombia; 9 Hemato Oncologos, Cali, Colombia; 10 Agencia Costarricense de Investigaciones Biomédicas (ACIB)-Fundación INCIENSA, Costa Rica; 11 Instituto de Nutrición y de Tecnología de los Alimentos, Santiago, Chile; 12 National Institute of Cancer, Santiago, Chile; 13 Section of Nutrition and Metabolism, International Agency for Research on Cancer, Lyon, France; 14 Division of Public Health Sciences, Fred Hutchinson Cancer Research Center, Seattle, United States of America; 15 Division of Human Biology, Fred Hutchinson Cancer Research Center, Seattle, United States of America; German Cancer Research Center (DKFZ), GERMANY

## Abstract

**Background:**

In Latin America (LA), there is a high incidence rate of breast cancer (BC) in premenopausal women, and the genomic features of these BC remain unknown. Here, we aim to characterize the molecular features of BC in young LA women within the framework of the PRECAMA study, a multicenter population-based case–control study of BC in premenopausal women.

**Methods:**

Pathological tumor tissues were collected from incident cases from four LA countries. Immunohistochemistry (IHC) was performed centrally for ER, PR, HER2, Ki67, EGFR, CK5/6, and p53 protein markers. Targeted deep sequencing was done on genomic DNA extracted from formalin-fixed, paraffin-embedded tumor tissues and their paired blood samples to screen for somatic mutations in eight genes frequently mutated in BC. A subset of samples was analyzed by exome sequencing to identify somatic mutational signatures.

**Results:**

The majority of cases were positive for ER or PR (168/233; 72%), and 21% were triple-negative (TN), mainly of basal type. Most tumors were positive for Ki67 (189/233; 81%). In 126 sequenced cases, *TP53* and *PIK3CA* were the most frequently mutated genes (32.5% and 21.4%, respectively), followed by *AKT1* (9.5%). *TP53* mutations were more frequent in HER2-enriched and TN IHC subtypes, whereas *PIK3CA/AKT1* mutations were more frequent in ER-positive tumors, as expected. Interestingly, a higher proportion of G:C>T:A mutations was observed in *TP53* in PRECAMA cases compared with TCGA and METABRIC BC series (27% vs 14%). Exome-wide mutational patterns in 10 TN cases revealed alterations in signal transduction pathways and major contributions of mutational signatures caused by altered DNA repair pathways.

**Conclusions:**

These pilot results on PRECAMA tumors give a preview of the molecular features of premenopausal BC in LA. Although the overall mutation burden was as expected from data in other populations, mutational patterns observed in *TP53* and exome-wide suggested possible differences in mutagenic processes giving rise to these tumors compared with other populations. Further -omics analyses of a larger number of cases in the near future will enable the investigation of relationships between these molecular features and risk factors.

## Introduction

Breast cancer (BC) incidence is increasing sharply in countries in economic transition, with a large number of cases in premenopausal women. In Latin America (LA), the proportion of BC in women younger than 45 years is nearly twice the proportion in developed countries, a difference that is only partly explained by population age structure [1]. Behavioral, reproductive, and lifestyle factors typical of Western populations are becoming more prevalent in LA and may play a role in the increased BC incidence in this population, but the reason for the sharp increase in incidence in premenopausal women in LA remains to be established [2].

BC is a heterogeneous disease in terms of biology and outcome. It is clinically classified into four subtypes (luminal A, luminal B, HER2-positive, and triple-negative [TN]), based on the expression of the estrogen receptor (ER), the progesterone receptor (PR), the human epidermal growth factor receptor 2 (HER2), and the proliferation marker Ki67 [3]. More sophisticated classifications based on genomic and transcriptional analyses provide a better description of the tumor biology and outcome [4, 5]. The two most frequently somatically mutated genes in BC are *TP53* and *PIK3CA* [6]. Mutations in *PIK3CA*, which render cells dependent on *PI3K* pathway signaling, are the most common genetic abnormality identified in hormone receptor-positive BC, whereas mutations in the tumor suppressor gene *TP53* are more prevalent in the HER2-enriched and TN subtypes [6–8].

Genomic analyses can also provide information related to tumor etiology. Indeed, somatic mutational signatures can reveal the contribution of specific mutational processes to the development of cancer. For example, *TP53* mutation patterns specific to exposure to exogenous mutagens have been reported in several cancer types [9], and at the genome-wide level, more than 30 mutational signatures have been described in cancer tissues and some have been linked to endogenous mechanisms of mutagenesis or to exposure to human carcinogens [10, 11].

Although BC genomic subtypes have been associated with different patient outcomes, how specific genomic alterations relate to risk factors or etiology remains largely unknown. Moreover, knowledge of the genomic features of premenopausal BC (preBC), particularly in countries in economic transition, is limited. The PRECAMA study was initiated to investigate the molecular, pathological, and risk factor patterns of preBC in LA (http://precama.iarc.fr/). It is the largest case–control study conducted in four countries in LA that systematically collects extensive information on lifestyle and risk factors as well as different biological samples (tumor tissues, blood fractions, and urine) according to standardized procedures. PRECAMA is thus a powerful framework for investigating relationships between BC tumor biology and etiology.

Here, we investigate the tumor genomic features of preBC in women in LA using the first set of samples collected within the framework of the PRECAMA study.

## Materials and methods

### Study population

The present study included 126 cases recruited between August 2012 and November 2015 in the context of the PRECAMA case–control study (http://precama.iarc.fr). Subjects included in PRECAMA are women diagnosed with BC at age 20–45 years and recruited at major general or cancer-dedicated hospitals in Chile, Colombia, Costa Rica, and Mexico that cover populations with a wide range of socioeconomic status. Women who had a positive biopsy for BC were recruited before any treatment. Women were invited to a home or hospital visit, during which a trained nurse presented the informed consent, collected biological samples and anthropometric measurements (height, weight, and hip and waist circumferences), and administered a standardized questionnaire on clinical, reproductive, and lifestyle risk factors. All participants gave written informed consent before enrollment, and the study protocols were approved by the institutional review boards of Chile (Oncologic Institute Foundation Arturo Lopez Pérez and National Cancer Institute), Colombia (Cancer Institute Las Americas and University of Antioquia), Costa Rica (Costa Rican Institute of Clinical Research [ICIC] and Center for Strategic Development and Information in Health and Social Security [CENDEISSS] of the Costa Rican Social Security Fund [CCSS]), Mexico (National Institute of Public Health and the Mexican Social Security Institute), and the International Agency for Research on Cancer (IARC).

### Biological specimens

Each study site applied common standardized protocols for specimen collection. The protocols were previously developed and extensively used by IARC [12, 13], and were subsequently fine-tuned based on a detailed review of the conditions at each center. Blood samples were obtained at recruitment by venipuncture using vacutainers, and buffy coats were prepared and stored at −80°C less than 6 hours after the blood draw. Buffy coats were shipped to IARC for genomic DNA extraction. Tumor samples were formalin-fixed and paraffin-embedded (FFPE) according to standard operating procedures. Paraffin blocks and hematoxylin and eosin sections were stored at the local pathology service facilities. Sections from tumor tissues were sent to Fred Hutchinson Cancer Research Center for centralized immunohistochemistry (IHC) analyses and tumor DNA extraction.

### Pathology review and IHC analyses

Histology sections from tumor biopsies obtained before any treatment were reviewed for histological diagnosis and grade, lymphovascular invasion, and stromal and lymphocyte response. IHC was conducted for ER (SP1, LabVision, Fremont, CA), PR (PgR 636, Dako, Denmark), HER2 (AO485, Dako, Denmark), epidermal growth factor receptor (EGFR) (31G7, Invitrogen, Camarillo, CA), CK5/6 (D5/16 B4, Dako, Denmark), p53 (Pab 1801, Calbiochem, La Jolla, CA), and Ki67 (MIB-1, Dako, Denmark) according to standardized and optimized protocols that included antigen retrieval when required. BCs were classified into subtypes according to ER, PR, and HER2 IHC results. Triple-negative (ER-, PR-, HER2-) BCs were additionally subtyped using EGFR and CK5/6 staining to define basal-like cancers. ER and PR positivity were defined as staining score >1%, and Ki67 positivity as staining >14%, as recommended by the St Gallen International Breast Cancer Conference [3].

### DNA extraction and sequencing

Tumor genomic DNA was extracted from 3–9 sections of 6 μm using the QIAamp DNA FFPE Tissue Kit (Qiagen) following the manufacturer’s recommended protocol, with the following modification. The tissue was incubated in ATL buffer and proteinase K overnight at 56°C with agitation, with the addition of 20 μL of proteinase K after the first 4 hours. Matched constitutive genomic DNA from cases was isolated from buffy coats at IARC with the Autopure LS system (Qiagen) using the "frozen buffy coat" protocol and following the manufacturer’s instructions. DNA was quantified by PicoGreen (ThermoFisher Scientific).

For **targeted sequencing**, exonic regions of the selected gene panel (*AKT1*, *CDH1*, *ERBB2*, *NOTCH1*, *PIK3CA*, *PTEN*, *RB1*, and *TP53*) were amplified from 80 ng of genomic DNA using GeneRead DNAseq Mix-n-Match Panel V2 (Qiagen) following the manufacturer’s instructions. Libraries were prepared with NEBNext reagents (New England BioLabs) following the manufacturer’s instructions. Libraries were quantified by PicoGreen (ThermoFisher Scientific) and pooled in equal quantities, and the library pool was quantified by the Qubit fluorometer (ThermoFisher Scientific) and quality checked with the Bioanalyzer (Agilent Technologies). Then, 800 pM of the library pool was used for sequencing on a Ion Proton sequencer (Life Technologies) according to the manufacturer’s instructions, aiming at a minimum of 100X coverage for blood DNA and 1000X coverage for tumor DNA. Tumor samples were processed in duplicate to control for artefactual mutations from FFPE fixation (see bioinformatics analyses below).

For **whole-exome sequencing**, exonic regions and splice junctions of tumor–blood DNA sample pairs were captured using the SeqCap EZ MedExome kit (Roche Diagnostics France) following the manufacturer’s instructions. This assay captures exonic regions covering 47 Mb of protein-coding bases. Libraries were prepared with the KAPA Hyper Prep Kit (Roche Diagnostics France) following the manufacturer’s instructions, and sequenced by 150-base paired-end massively parallel sequencing on an Illumina HiSeq 4000 sequencer at the New York University Langone Medical Center according to the manufacturer’s instructions.

### Bioinformatics analyses

Data from the Ion Proton sequencer were processed with the Ion Torrent built-in pipeline (TorrentSuite V4) to generate BAM files, and variant calling was done with the built-in ITVC in the somatic mode and with a minimum allele frequency threshold of 4%. Variants were annotated with Annovar and filtered to eliminate known single nucleotide polymorphisms (SNPs) (variants present in the Exome Aggregation Consortium [ExAC] or 1000 Genomes [1000G] databases at a frequency >0.001) and sequencing artefacts using the MutSpec Galaxy package developed in-house [14]. Further manual checks of BAM files using IGV were done when appropriate. All non-synonymous mutations found in the targeted regions and present in both duplicates of tumor samples but not in any blood samples of the 126 cases were retained for analysis.

Exome data from the HiSeq 4000 sequencer were analyzed with a pipeline developed in-house and based on standard tools for quality control and processing (FastQC 0.11.3, AdapterRemoval 2.1.7, BWA-MEM 0.7.15, Qualimap 2, GATK 3.5, and Picard 1.131). Somatic variant calling was done on tumor–blood sample pairs with Strelka [15] using the default parameters. Variant annotation and filtering was done as described above with MutSpec [14], and only somatic indels and single nucleotide variants (SNVs) in coding regions were retained and analyzed. Pathway analysis of mutated genes was done with ConsensusPathDB (r32) using the KEGG, Biocarta, Reactome, and WikiPathways databases and a minimum of 3 overlapping genes and *q*-value <0.05 as settings [16]. To define cancer genes, we used the Catalogue of Somatic Mutations in Cancer (COSMIC) Cancer Gene Census (v82) [17], and genes identified as drivers for BC in the IntOGen database (r2014.12) [18].

### Public data on somatic mutations in breast cancer

Data from The Cancer Genome Atlas (TCGA) breast and METABRIC genomic studies [19, 20] and from the IARC TP53 Database [21] were used as comparison datasets. Gene-specific mutation files (*AKT1*, *CDH1*, *ERBB2*, *NOTCH1*, *PIK3CA*, *PTEN*, *RB1*, and *TP53*) and related clinical files for the TCGA and METABRIC studies were retrieved from cBioPortal [22, 23] in February 2017. MAF files from exome sequencing data of TCGA BC cases were retrieved on 26 March 2015 via a https protocol at https://tcga-data.nci.nih.gov/tcgafiles/ftp_auth/distro_ftpusers/anonymous/tumor/. Gene-specific data from TCGA and METABRIC were combined, including only cases with documented age and ER, PR, and HER2 status, and stratified by age (younger than 45 or older than 55 years). For TCGA exome data, only data with documented age and ER, PR, and HER2 status were selected, resulting in a dataset of 453 samples, including 96 preBC and 357 postBC. Version R18 of the somatic dataset of the IARC TP53 Database was used to select for mutations reported in primary BC in women age 45 or younger and in studies using Sanger sequencing. Finally, another independent dataset, named hereafter 560BC, was assembled from public data obtained from whole-genome sequencing of 560 BC cases [6]. For this dataset, mutation data were retrieved from COSMIC and clinical data were retrieved from the original publication. Only cases with documented ER, PR, and HER2 status and diagnosed at 45 years or younger were included (*N* = 123).

### Statistical analyses

Associations between study variables were tested using Fisher’s exact test. For mutational signature analyses, we used PRECAMA exome data (*N* = 12 samples) and TCGA exome data (*N* = 453). Mutations were classified into 96 types, corresponding to the 6 possible base substitutions (C:G>A:T, C:G>G:C, C:G>T:A, T:A>A:T, T:A>C:G, and T:A>G:C) and the 16 possible pairs of nucleotides immediately flanking 5′ and 3′. Mutational signatures in these samples were then extracted using the non-negative matrix factorization (NMF) algorithm implemented in a NMF R package [24, 25]. NMF decomposition identifies signatures and estimates their contributions to each sample. Six signatures were identified using the cophenetic correlation coefficient as a measure of stability of the signatures. We calculated the cosine similarity between the 6 extracted signatures and those published in COSMIC and in other original reports [17, 26], as described elsewhere [27].

We wished to identify possible systematic differences of signature contributions between IHC subtypes, by menopausal status, and by study source (TCGA vs PRECAMA). Because of the small number of PRECAMA samples, we used 2000 permutations of samples to obtain an empirical distribution of the Kruskal–Wallis rank-sum statistic. This permutation test was applied to test for possible association of each signature with a) menopausal status; b) IHC subtype; c) menopausal status stratified by IHC subtype; d) menopausal status adjusted for subtype by linear model; and e) study source, with partial adjustment for subtype (TN vs others) and also restricted to preBC samples.

All statistical analyses were performed using R statistical software version 3.3.2. The statistical significance level was set to 0.05, without adjustment for multiple comparisons.

## Results

### IHC subtypes in PRECAMA tumors

In the first consecutive cases recruited in PRECAMA, for which tumor pathological evaluation has been completed (*N* = 229), most BC cases (72%) were ER-positive and 16% were HER2-positive (**Table 1**). Using ER/PR/HER2 IHC subtyping, the majority of cases were luminal A (58%), followed by TN (21%), luminal B (11%), and HER2-enriched (5%) (**Table 1**). TN tumors were predominately basal-like (94%) (**Table A in S1 File**). Proliferation status was assessed by Ki67 IHC staining. More than 80% (189/233) of cases had high Ki67 staining with a median percentage of 31.6 (not shown). Overall, Ki67-positivity (staining >14%) was significantly associated with IHC subtypes (*p*-value = 2 × 10^−4^; Fisher’s exact test). In particular, the proportion of Ki67-positive cases was significantly lower in luminal A cases than in TN cases (72% vs 98%, *p*-value = 2.8 × 10^−5^).

**Table 1:** Sample classification by IHC results.

**IHC result**	**Number of samples** ***N* (%)**	**Ki67-positive** ***N* (%)**
**ER**		
**Negative**	65 (28%)	63 (97%)
**Positive**	168 (72%)	126 (75%)
**PR**		
**Negative**	71 (30%)	67 (94%)
**Positive**	162 (70%)	122 (75%)
**HER2**		
**Negative**	183 (79%)	144 (79%)
**Equivocal**	13 (6%)	12 (92%)
**Positive**	37 (16%)	33 (90%)
**Total**	233	189 (81%)
IHC SUBTYPE*		
**Luminal A**	134 (58%)	96 (72%)
**Luminal B**	26 (11%)	23 (88%)
**HER2-enriched**	11 (5%)	10 (91%)
**Triple-negative**	48 (21%)	47 (98%)
***Of basal type***	*45 (94%)*	*42 (93%)*
Undetermined**	14 (6%)	13 (93%)

* Tumor subtype definitions: luminal A: ER+/HER2-; luminal B: ER+/HER2+; HER2-enriched: ER-/HER2+; triple-negative: ER-/PR-/HER2-; TN of basal type: EGFR+ and/or CK5/6+.

** 14 cases were not assigned a subtype: 13 cases had equivocal HER2 results and no confirmatory FISH; 1 case was ER-/PR+/HER2- with a weak PR positivity.

### Somatic mutations in premenopausal BC

**Fig 1:**
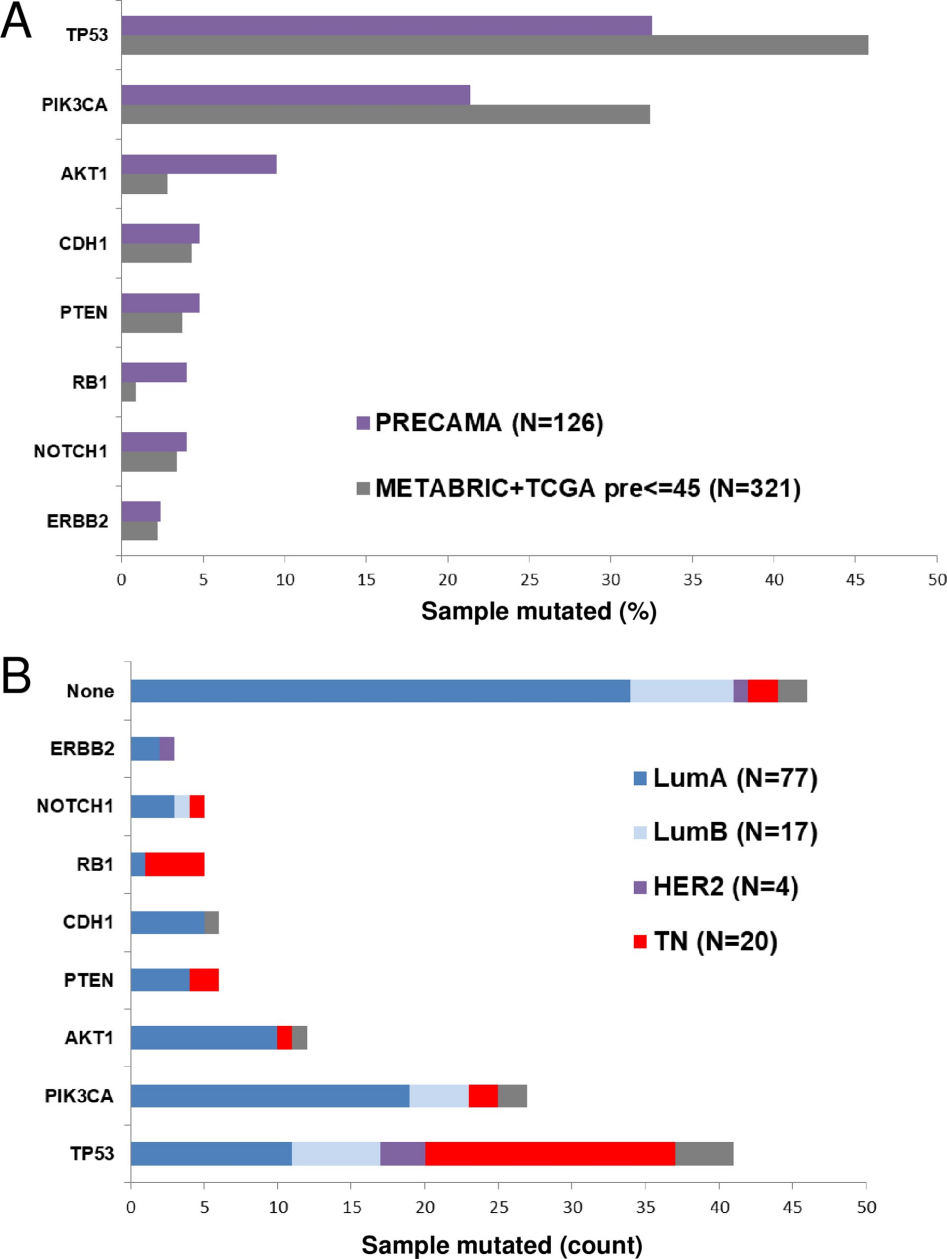
Occurrences of mutations in 8 BC genes. (A) Gene mutation frequencies in PRECAMA samples are compared with those observed in a dataset of premenopausal women selected from the TCGA and METABRIC BC series [19, 20]. (B) IHC subtype distributions of samples according to their mutation status. Luminal A: ER+/HER2-; luminal B: ER+/HER2+; HER2-enriched: ER-/HER2+; triple-negative: ER-/PR-/HER2-.

Tumor genomic DNA was extracted from FFPE tissue sections prepared at each collecting center according to a standardized protocol. More than 250 ng of DNA was obtained for 75% of the samples, with a median yield of 994 ng. A limiting amount of DNA (<100 ng) was obtained for 12% (21/172) of the samples. Targeted deep sequencing of a panel of 8 genes frequently mutated in BC (*AKT1*, *CDH1*, *ERBB2*, *NOTCH1*, *PIK3CA*, *PTEN*, *RB1*, and *TP53*) was successfully performed on 126 cases for which more than 250 ng of tumor genomic DNA was available. Tumor DNA and patient-matched blood DNA were sequenced at minimum coverages of 1000X and 100X, respectively. To control for potential artefacts due to formalin fixation, FFPE tumor samples were sequenced in duplicates and only mutations detected in both duplicates were considered (see Materials and Methods). Potentially deleterious somatic mutations (affecting splicing, indels, nonsense, stop-loss, and non-synonymous substitutions) in the 8-gene panel were found in 63.5% (80/126) of samples. *TP53* was the most frequently mutated gene (32.5%), followed by *PIK3CA* (21.4%) and *AKT1* (9.5%), whereas other genes were mutated in less than 5% of samples. This distribution differed from that observed in preBC from the TCGA/METABRIC datasets (**Fig 1A**). Indeed, there were fewer cases with *TP53* or *PIK3CA* mutations and more cases with *AKT1* and *RB1* mutations in PRECAMA versus TCGA/METABRIC cases (*p*-values at best = 0.03). These differences may be explained in part by a different distribution of subtypes between PRECAMA and TCGA/METABRIC cases (*p*-value = 8.2 × 10^−6^), because a higher proportion of luminal A cases (known to carry frequent *AKT1* and infrequent *TP53* mutations) and a lower proportion of TN cases (known to carry frequent *TP53* mutations) was observed in PRECAMA compared with TCGA/METABRIC (*p*-value = 0.04). Interestingly, whereas the *PIK3CA*/*AKT1* pathway was mutated at the expected rates in the luminal A PRECAMA tumors, *AKT1* mutations were more frequent relative to *PIK3CA* mutations in PRECAMA compared with TCGA/METABRIC luminal A cases (14% *AKT1* and 23% *PIK3CA* mutations in PRECAMA vs 4% *AKT1* and 54% *PIK3CA* mutations in TCGA/METABRIC), although this was not statistically significant.

*PIK3CA* and *AKT1* mutations were at classical hotspots (p.H1047R, p.E542K, and p.E545K for *PIK3CA* and p.E17K for *AKT1*), and *TP53* mutations were mostly missense substitutions that spread across the coding sequence (**Table A in S1 File**). The relationship between IHC subtypes and mutated genes was as expected from previous studies (**Fig 1B**). *TP53*, *RB1*, or *PTEN* mutated samples had higher proportions of the TN subtype, whereas *AKT1* or *CDH1* mutated samples had higher proportions of the luminal A subtype. The majority of samples with no mutation in the tested genes were of luminal A subtype (34/46; 74%).

Twenty one tumors had mutations in more than one gene (**Table A in S1 File** filtered for genes_mutated >1). One case was of HER2-enriched subtype and had mutations in *TP53* and *ERBB2*. Three cases were of luminal B subtype and had mutations in *PIK3CA* and *TP53* or *CDH1*. Seven cases were of TN subtype and had mutations in *TP53* combined with *RB1* (3 cases), *PTEN* (2 cases), *PIK3CA* (1 case), or *NOTCH1* (1 case). Ten cases were of luminal A subtype and had mutations in *TP53* and *PIK3CA* (4 cases), in *TP53* and *AKT1* (3 cases), or in other gene combinations. Details of mutations are provided in **Table A in S1 File**.

**Fig 2:**
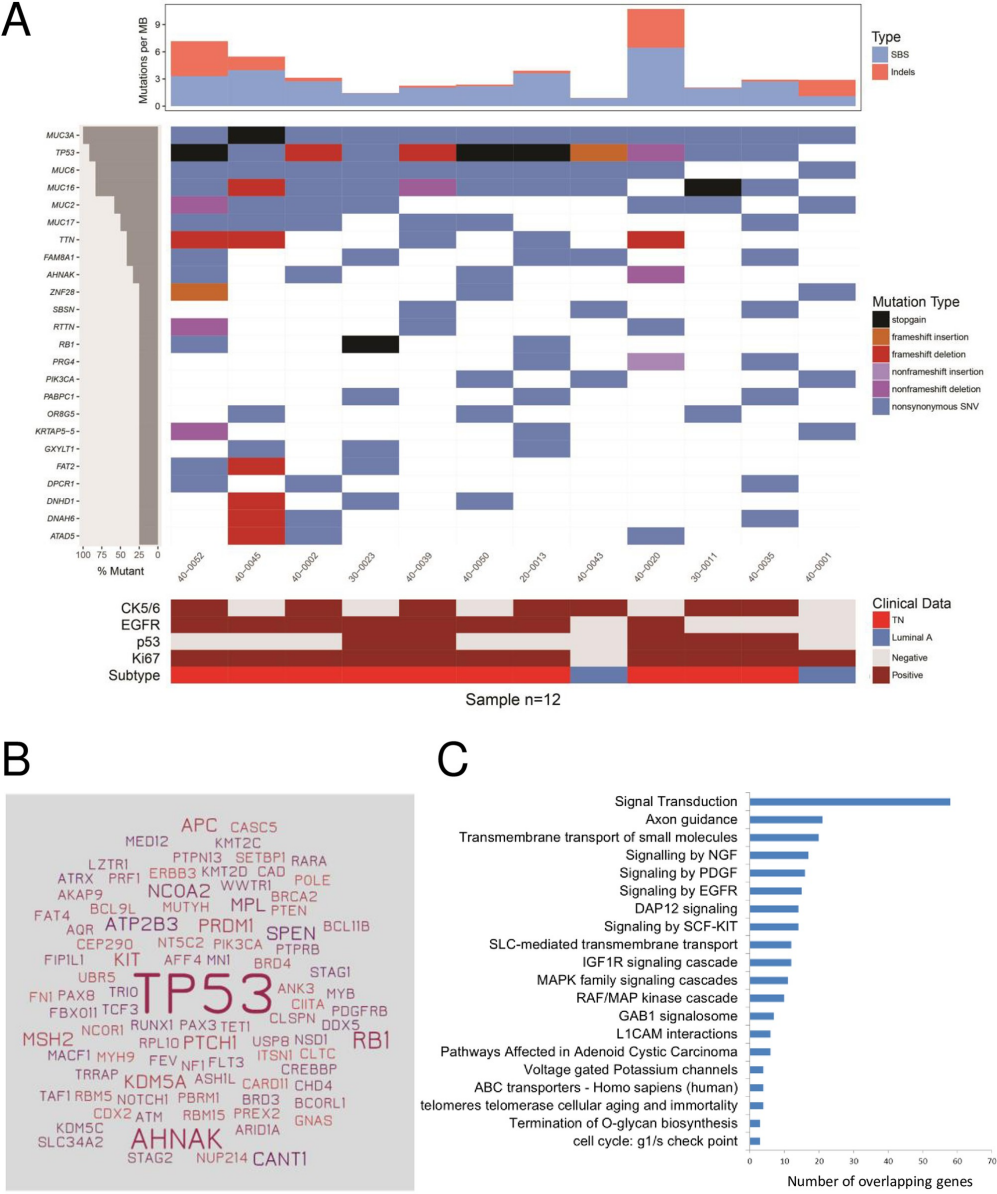
Whole-exome sequencing results in 12 PRECAMA samples. Only coding non-silent somatic mutations are considered. (A) Mutation rates (top panel), top mutated genes and their mutation types (middle panel), and IHC features (lower panel), sorted by top mutated genes. Luminal A: ER+/HER2-; triple-negative: ER-/PR-/HER2-. (B) All cancer genes somatically mutated in the 10 TN samples are depicted; the size of gene names is proportional to the number of samples mutated for each gene. (C) Pathways enriched (*q*-value <0.05) in the list of genes mutated in TN cases with allele frequency >20% and predicted deleterious/probably deleterious by PolyPhen-2 (*N* = 333 genes). Number of overlapping genes in each pathway is shown.

In a subset of 12 samples (2 luminal A and 10 TN cases selected randomly) analyzed with the 8-gene panel, we also performed whole-exome sequencing. With a median coverage of 200X in tumor DNA and 80X in blood DNA and more than 99.5% of mapped reads (see **Table B in S1 File**), we identified 2634 somatic mutations in coding regions, including 2128 non-synonymous SNVs and indels (see **Table C in S1 File**). All mutations found by targeted sequencing in the 8-gene panel were confirmed in the exome analysis. There was an average of 3.9 non-synonymous SNVs and indels per MB, with 2 samples carrying more than 6 mutations per MB (**Fig 2A, top panel**). The top mutated genes included four cancer genes (*TP53*, *RB1*, *PIK3CA*, and *AHNAK*) and several large genes, such as mucin genes and the *TTN* gene (**Fig 2A, middle panel**). *AHNAK* has recently been described as a novel tumor suppressor gene in BC, especially in the TN subtype, acting via different signaling pathways, such as AKT/MAPK or TGFβ [28, 29]. The *AHNAK* mutations, like the *RB1* mutations, were all in TN cases. However, the impact of *AHNAK* mutations on protein function is unknown, because *AHNAK* is a large gene and 75% of the mutations were predicted as benign by PolyPhen-2 [30]. In TN cases (*N* = 10), there were 92 cancer genes mutated, dominated by *TP53*, which was mutated in all samples, and with 14 other cancer genes mutated in more than one sample (**Fig 2B**). Pathway enrichment analysis of potential driver mutations in these TN samples (**Table D in S1 File**) showed enrichment for several growth factor signaling pathways and for pathways involved in insulin receptor signaling, telomere maintenance, transmembrane transport of small molecules, and G1 checkpoint or O-glycan biosynthesis (**Fig 2C** and **Table E in S1 File**).

### Mutation patterns and signatures in premenopausal BC

**Fig 3:**
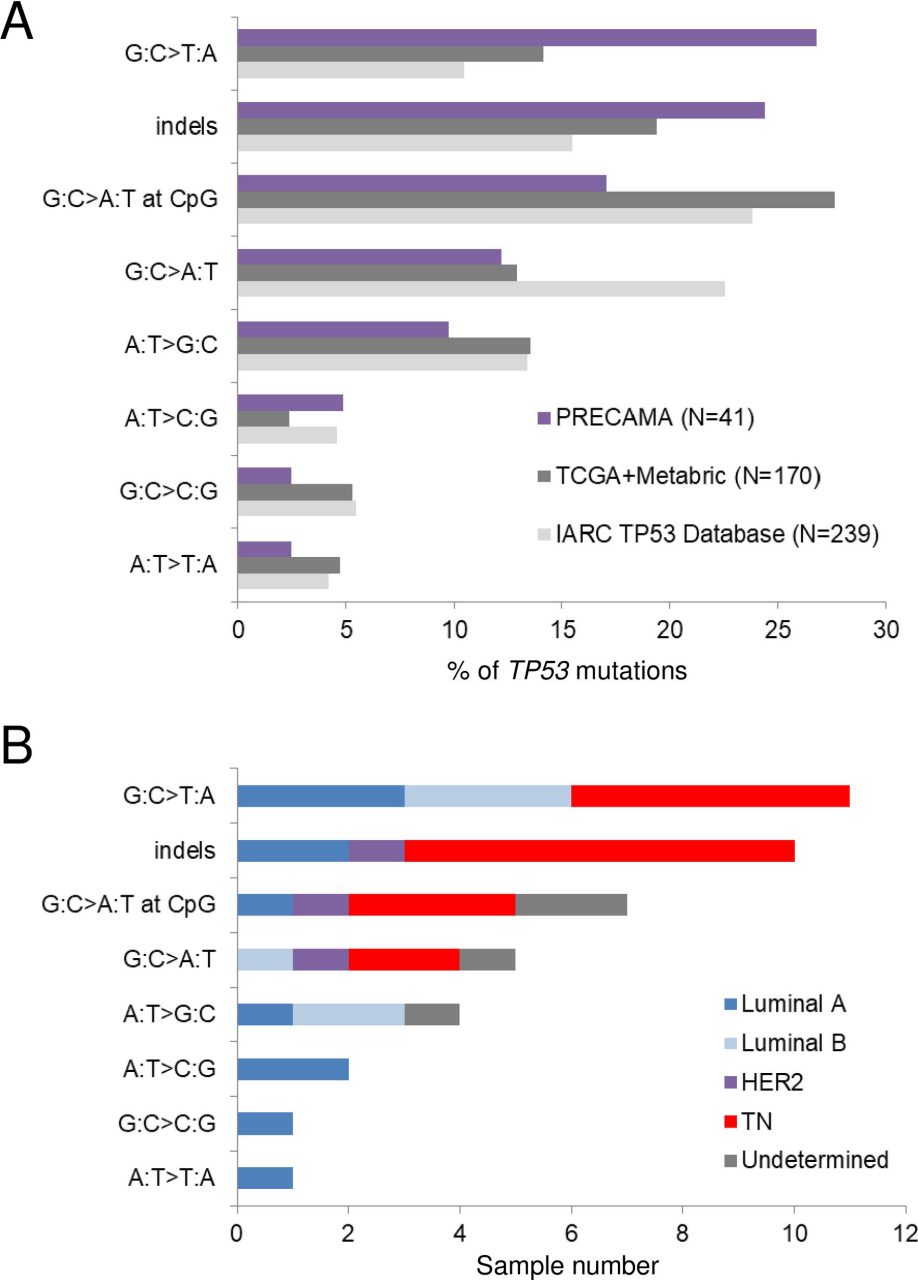
Distribution of *TP53* mutation types in preBC. (A) Distribution of mutation types in PRECAMA samples is compared with those observed in women 45 years old or younger selected from the TCGA and METABRIC BC series [19, 20] or the IARC TP53 Database [21]. (B) IHC subtype distributions of PRECAMA samples in each mutation type category. Luminal A: ER+/HER2-; luminal B: ER+/HER2+; HER2-enriched: ER-/HER2+; triple-negative: ER-/PR-/HER2-.

**Fig 4:**
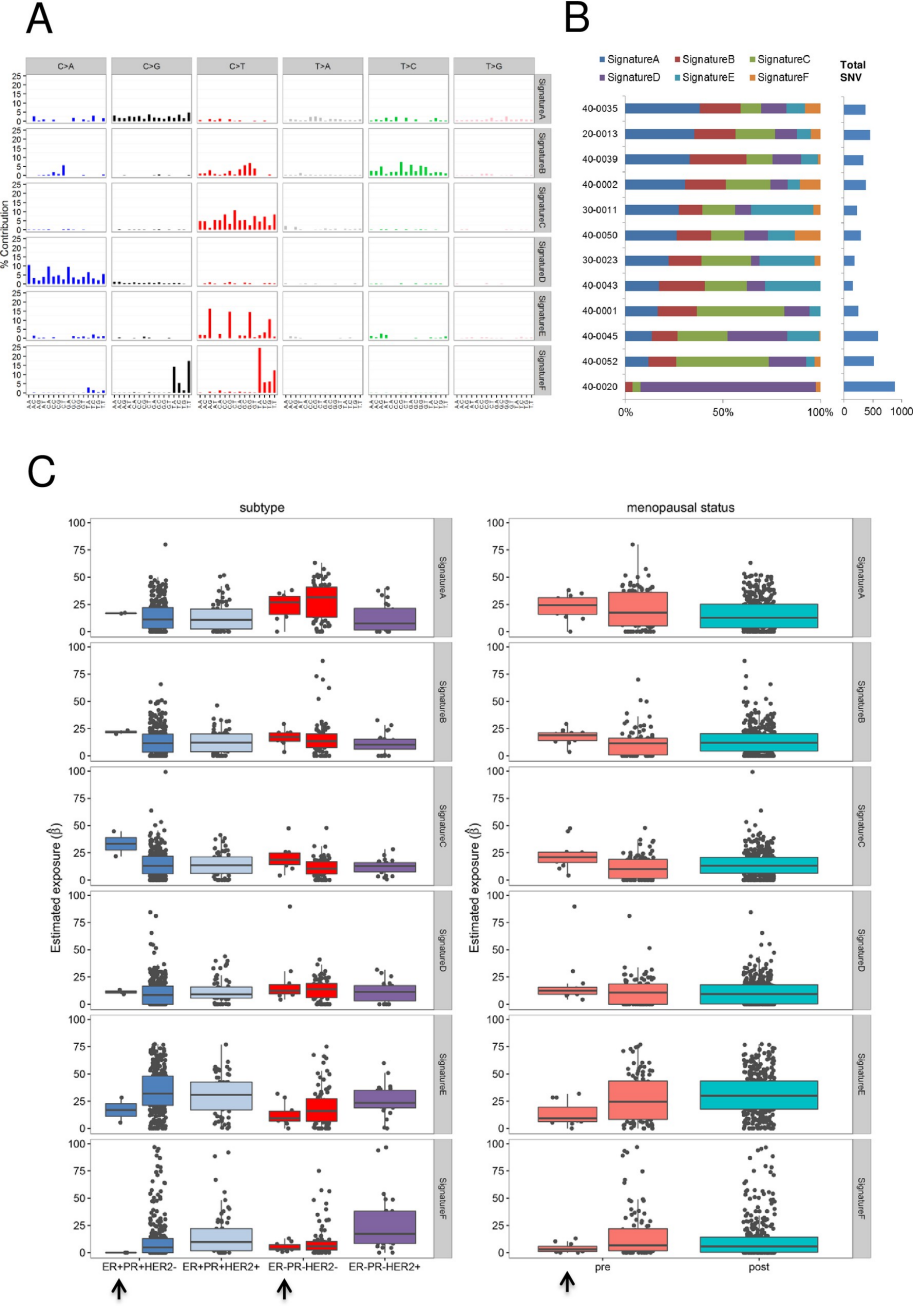
Mutational signatures identified in TCGA and PRECAMA samples, and their relationship with tumor subtype and patient menopausal status. (A) The 6 mutational signatures identified in 453 TCGA samples (including preBC and postBC) plus 12 PRECAMA samples. The 6 types of base substitutions are color-coded and further stratified by their adjacent 5′ and 3′ sequence context. Sig.A matches with COSMIC signature-3; Sig.B matches with COSMIC signature-26; Sig.C matches with COSMIC signatures-11/19/23/30 and experimental signatures of MNU and MNNG; Sig.D matches with COSMIC signature-18; Sig.E matches with COSMIC signature-1; and Sig.F matches with COSMIC signatures-2/13 (see **Table A in S1 File**). (B) Percentage contributions of the 6 mutational signatures to the SNVs found in PRECAMA samples. (C) Percentage contributions of the 6 mutational signatures in the PRECAMA and TCGA samples stratified by tumor IHC subtypes (left graphs) and by menopausal status (right graphs). PRECAMA samples are indicated with arrows.

To study the underlying mutational processes involved in the development of preBC tumors in the studied populations, we analyzed somatic mutation patterns in the *TP53* gene and at the exome-wide level. **Fig 3A** shows the distribution of *TP53* mutation types in PRECAMA tumors and in tumors from young women (< = 45 years) from other datasets. There was a higher proportion of G:C>T:A mutations in PRECAMA compared with the IARC TP53 Database (*p*-value = 0.004) or TCGA/METABRIC (*p*-value = 0.05) datasets. In fact, G:C>T:A was the most frequent type, followed by indels, in PRECAMA, whereas G:C>A:T at CpG was the most frequent type in the other datasets. The overall distribution of *TP53* mutation types was not significantly associated with IHC subtypes in PRECAMA samples (*p*-value = 0.06), although cases with indels were more frequently of the TN subtype (**Fig 3B**). *TP53* indels were truncating mutations (predicted to result in loss of p53 protein expression) in 6/10 cases, and 5/6 of these truncating mutations were indeed associated with null p53 IHC staining (see **Table A in S1 File**). Therefore, although the presence of frequent *TP53* truncating mutations in the TN subtype was similar to previous reports [31], the high frequency of G:C>T:A mutations in PRECAMA was unexpected. To validate this result, we used another dataset from a whole-genome sequencing study [6], and PRECAMA samples did show a significantly higher frequency of G:C>T:A mutations (**S2B Fig**) (*p*-value = 0.02). Mutational signatures at the exome-wide level were analyzed using a dataset including the 12 PRECAMA samples and 453 BC samples from TCGA (including both preBC and postBC cases; see Materials and Methods). We identified 6 signatures that matched with previously reported signatures (**Fig 4A and Table F in S1 File**). The estimated contribution of each signature to the mutation load in PRECAMA samples (**Fig 4B**) showed that 5/6 signatures had a contribution above 20% in at least one sample. Sig.A had the highest median contribution in these samples (24.3%). Sig.A matched with COSMIC signature-3, which has been established as a biomarker of homologous recombination defects through genetic and epigenetic inactivation of the *BRCA1/2* pathway, a distinctive feature of basal-like tumors [6, 10, 32]. Sig.B, which contributed in 6/12 samples, matched with COSMIC signature-26, proposed to be linked to defective DNA repair and previously reported in BC. Sig.C, which contributed in 6/12 samples, matched with several signatures characterized by C>T mutations outside CpG sites, including experimental signatures induced by alkylating agents (MNNG and MNU) in rodent systems [26, 27], COSMIC signature-11, observed in recurrent brain tumors of patients treated with MNNG [33], and COSMIC signature-30, of unknown origin but previously observed in some BC. Sig.D, which matched with COSMIC signature-18, was mainly observed in one sample, where it contributed to 90% of the mutation load and where the overall mutation load was the highest. The origin of this signature in BC remains to be established, but it has recently been associated with germline mutation in the repair enzyme *MUTYH* in colorectal and adrenocortical carcinomas [34, 35]. Interestingly, the sample in which Sig.D dominated carried a truncating somatic mutation in *MUTYH* (see **Table A in S1 File**). Finally, Sig.E, characterized by C>T mutations at a CpG site and matching with COSMIC signature-1, known to be due to spontaneous deamination of 5-methylcytosine (also referred as the “age” signature), had a contribution of at least 20% in only 3 samples.

As shown in **Fig 4C**, we explored possible systematic differences of signature contributions between IHC subtypes, and by menopausal status or study source (TCGA vs PRECAMA). Sig.F, which matched with COSMIC signature-2 and COSMIC signature-13, linked to mutagenesis by APOBEC, was more prevalent in HER2-enriched subtype cases and underrepresented in TN cases (*p*-value < 5 × 10^−4^, permutation test), as reported previously [36]. This is consistent with the fact that we did not find a strong contribution of the APOBEC signature in the PRECAMA samples (median contribution: 2.9%) because we analyzed only TN and luminal A cases. COSMIC signature-3 (Sig.A) was enriched in TN cases (*p*-value < 5 × 10^−4^) and preBC (*p*-value = 0.03). This signature was the predominant one in PRECAMA TN cases (median contribution: 26.8%). The contribution of the “age” signature (Sig.E) was lower in TN cases than in all other subtypes (*p*-value < 5 × 10^−4^) and also lower in preBC compared with postBC (*p*-value = 0.006). It was the lowest in PRECAMA samples (13.4% vs 30.5%, *p*-value = 1 × 10^−3^). The contribution of Sig.D was slightly higher in TN cases compared with all other subtypes (*p*-value = 0.01) and was the main contributor to the mutation load in one PRECAMA sample (**Fig 4B**). Because this sample carried a somatic mutation in *MUTYH*, and a recent study found germline mutations in *MUTYH* in young women with BC [37], it will be interesting to further study the role of *MUTYH* alteration in TN and preBC. In stratified analyses by IHC subtypes, the contributions of signatures in PRECAMA TN cases were similar to those observed in TCGA TN samples, except for Sig.C (contribution was higher in PRECAMA than in TCGA TN samples, *p*-value = 0.006; median contributions: 18.6% vs 10.4%). Because Sig.C matched with several signatures, including signatures linked to exposure to alkylating agents not expected in these treatment-naive samples, its origin remains to be established. There was no effect of menopausal status on the contributions of signatures when taking into account IHC subtype using linear models (all *p*-values > 0.16; permutation tests of linear regression model).

## Discussion

The results obtained in this pilot phase of the PRECAMA study demonstrate the feasibility of advanced genomic analyses of the tumor and blood samples collected at multiple sites in LA. They provide a preview of the molecular features of preBC in that population, with interesting mutational patterns that deserve further study.

Indeed, more than 92% of samples processed for IHC analyses were successfully scored for 7 markers (only 20/253 were excluded due to absence of invasive tumor or insufficient tissue for testing), and 80% of samples processed for DNA extraction yielded DNA quantities and quality compatible with genomic analyses (136/172 samples yielded more than 200 ng of DNA). With a target of 1200 cases recruited for the full study (with Guatemala and Brazil joining the study), this will be the largest series of preBC in Latin American women with genomic characterization of the tumors.

The IHC analyses showed a majority of ER-positive cases and a proportion of TN subtype similar to previous reports in Hispanic women [38]. The overall prevalence of ER-negative tumors in PRECAMA was substantiated by sequencing results on the 8-gene panel analyzed here. Indeed, *TP53* mutations, which are strongly associated with ER-negative status [7, 39], were found in 32.5% of the cases, consistent with an overall 28% of ER-negative cases. Also, the frequency of *AKT1* mutations, typical of ER-positive cases [20, 40], was higher in PRECAMA than in the comparative dataset of young women. Continued enrollment will enable us to determine more precise estimates of subtype distribution in PRECAMA and to explore potential differences in tumor subtype distributions between countries.

Although the overall tumor characteristics were more similar to those described in postBC than in preBC from other series, IHC staining with Ki67 showed high levels of staining in these preBC samples, even in luminal A cases (72% positive cases), which is consistent with previous reports on preBC [41, 42]. Liao et al. (2015) [43] recently compared the molecular features of preBC versus postBC from the TCGA and METABRIC datasets using multi-omic data integration. They reported no difference in gene expression between preBC and postBC in ER-negative cases but significant differences in ER-positive cases, with activation of integrin signaling and EGFR pathways and TGFβ as the top upstream regulator in preBC. It would therefore be important in future studies to assess whether activation of these pathways drives the level of proliferation reflected by high Ki67 positivity in ER-positive preBC, because they may be potential clinical targets.

The characteristics of the mutations found by target sequencing of the 8-gene panel were similar to those observed in other series of BC, with classical hotpots found in *AKT1* and *PIK3CA*, a majority of missense mutations found in *TP53*, a higher proportion of truncating *TP53* mutations in TN cases compared with other subtypes, and an expected distribution of mutated genes within IHC subtypes. However, an interesting difference in the distribution of *TP53* single base substitutions was observed. The most frequent *TP53* mutation type was G:C>T:A, which represented 27% of all *TP53* mutations. This proportion of G:C>T:A mutations was 1.5–3.3 times those observed in the comparative datasets used here, matching figures reported in lung cancers linked to exposure to polycyclic aromatic hydrocarbons [44, 45]. This pattern is therefore unexpected in BC. These G:C>T:A mutations do not exhibit a strand bias, do not cluster at any hotspot, and seemed similarly distributed within IHC subtypes or country of origin, although the numbers are still too low to enable any conclusion to be drawn. Because these results may suggest a specific, as-yet unknown, mutational process at the origin of *TP53* mutations, it will be important to confirm them in the full PRECAMA study.

Exome-wide mutation profiling of a subset of basal-like TN tumors confirmed that *TP53* and *RB1* were the only cancer genes recurrently affected by deleterious mutations (>2 samples). These results are concordant with previous reports on TNBC of basal-like type that showed a predominance of *TP53* mutations and of *TP53* and *RB1* pathway alterations [40, 46]. These reports also suggest activation of the PIK3CA/AKT pathway, based on gene copy number analyses (*PIK3CA* gene amplification, *PTEN* gene deletion) and protein phosphorylation assays [40]. Here, we found one activating *PIK3CA* mutation in 10 TNBC samples, which is in the range of previous reports (9%). However, because we limited our analyses to SNVs and small indels, we could not further assess the functionality of the *PIK3CA* pathway. Pathway analysis of potentially functional mutations across all genes showed enrichment of signal transduction pathways including EGFR, PDGF, and IGF1R, and mutational signatures showed a large contribution of DNA repair defects to the mutation load. These overall results on TN cases are consistent with our previous analyses of another series of TN cases from Mexico, in which transcriptomics analyses showed an overexpression of growth-promoting signals (including *EGFR*, *PDGFR*, and *PIK3CA*), a repression of cell cycle control pathways (*TP53* and *RB1*), and a deregulation of DNA repair pathways [47].

Our exploratory analysis of exome-wide mutational signatures in relation to IHC subtype and menopausal status in the TCGA and PRECAMA samples showed that the contributions of mutational signatures are determined by the tumor subtype but not the menopausal status, and that PRECAMA TN cases showed contributions similar to TCGA TN samples for 5/6 signatures identified in the analyzed set.

Some limitations of the results presented should be noted. First, the prevalences of IHC subtypes are based on still-limited numbers and may therefore not be representative of the distribution at the population level. Second, confirmation of HER2 status by FISH could not be done in this pilot phase, and therefore the prevalence of the luminal B or HER2-enriched subtypes may be under- or over-estimated. Third, the exome analyses have been performed on a limited number of cases to establish the feasibility of these assays. Results on this small set did show feasibility and enabled us to identify both similarities and differences in genomic alterations compared with other series of BC. Analysis of the full series will determine whether any specific genomic feature may characterize preBC in women in LA.

## Conclusions

These pilot results on PRECAMA tumors give a preview of the molecular features of preBC in LA. Although the overall mutation burden was as expected from data in other populations, mutational patterns observed in *TP53* suggested possible differences in mutagenic processes giving rise to these tumors compared with other populations. Further -omics analyses of a larger number of PRECAMA cases in the near future will enable the investigation of relationships between these molecular features and etiological factors.

## Supporting information

S1 File **Tables A-F** Table A: Demographics and molecular characteristics of cases analyzed by next-generation sequencing; Table B: Whole-exome sequencing data metrics; Table C: Mutations in coding regions from whole-exome sequencing and mutation calling with Strelka; Table D: List of mutated genes in TN cases with mutations present at an allele frequency >20% and predicted to affect protein function (splice, truncating, and non-synonymous predicted deleterious/probably deleterious by PolyPhen-2) (*N* = 333); Table E: Pathway analysis of 333 altered genes in TN samples; Table F: Cosine similarity values for the comparisons between each of the 6 extracted signatures and 37 published signatures.
(XLSX)

S1 Fig Distribution of IHC subtypes in PRECAMA samples and in preBC extracted from METABRIC and TCGA studies. Comparison of the distribution of IHC subtypes observed in PRECAMA and in preBC from a dataset extracted from METABRIC and TCGA (see Materials and Methods). Luminal A: ER+/HER2-; luminal B: ER+/HER2+; HER2-enriched: ER-/HER2+; triple-negative: ER-/PR-/HER2-.
(PDF)

S2 Fig Mutation characteristics and distribution of IHC subtypes in PRECAMA samples compared with an independent dataset of preBC. Data on 123 preBC cases with receptor status information reported in Nik-Zainal et al. (2016) were retrieved from supplementary materials (clinical information) or from COSMIC (mutation data) [6]. (A) Occurrences of mutations in the 8 BC genes analyzed in PRECAMA. (B) Distribution of *TP53* mutation types in preBC cases. (C). Comparison of the distribution of IHC subtypes observed in preBC in the two datasets. Luminal A: ER+/HER2-; luminal B: ER+/HER2+; HER2-enriched: ER-/HER2+; triple-negative: ER-/PR-/HER2-.
(PDF)

## Abbreviations

BC: breast cancer
COSMIC: Catalogue of Somatic Mutations in Cancer
EGFR: epidermal growth factor receptor
ER: estrogen receptor
FFPE: formalin-fixed, paraffin-embedded
HER2: human epidermal growth factor receptor 2
IHC: immunohistochemistry
LA: Latin America
postBC: postmenopausal breast cancer
PR: progesterone receptor
preBC: premenopausal breast cancer
SNVs: single nucleotide variants
TCGA: The Cancer Genome Atlas
TN: triple-negative
TNBC: triple-negative breast cancer.
