# Supplementary figures and images for: Molecular features of premenopausal breast cancers in Latin American women: Pilot results from the PRECAMA study
Source: PLoS One. 2019 Jan 17;14(1):e0210372. doi: 10.1371/journal.pone.0210372 (PMC6336331; doi:10.1371/journal.pone.0210372)

S1 Fig

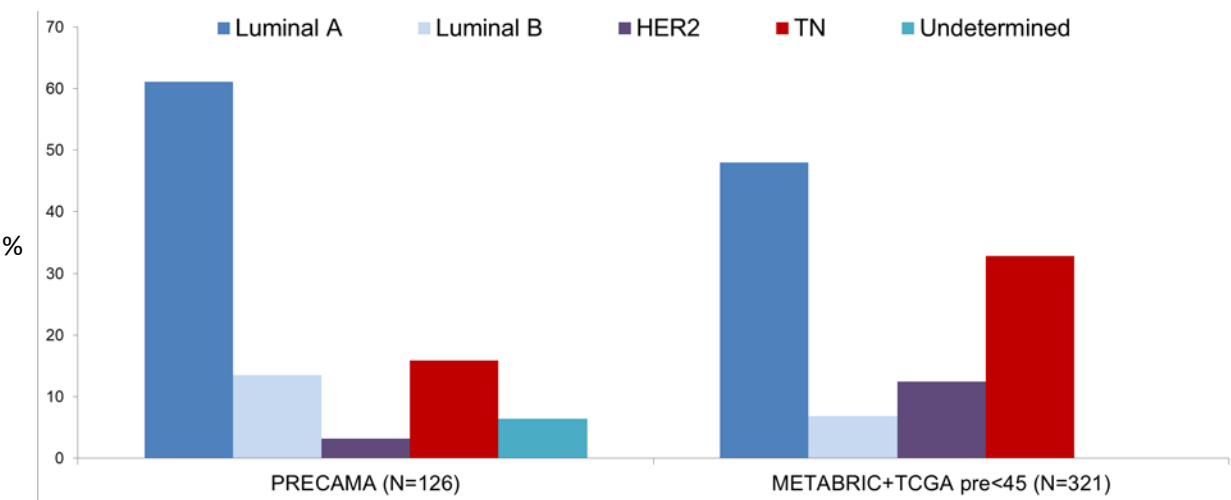

Supplement: S1 Fig — Comparison of the distribution of IHC subtypes observed in PRECAMA and in preBC from a dataset extracted from METABRIC and TCGA (see Materials and Methods). Luminal A: ER+/HER2-; luminal B: ER+/HER2+; HER2-enriched: ER-/HER2+; triple-negative: ER-/PR-/HER2-. (PDF) [file pone.0210372.s002.pdf]

S2 Fig

A

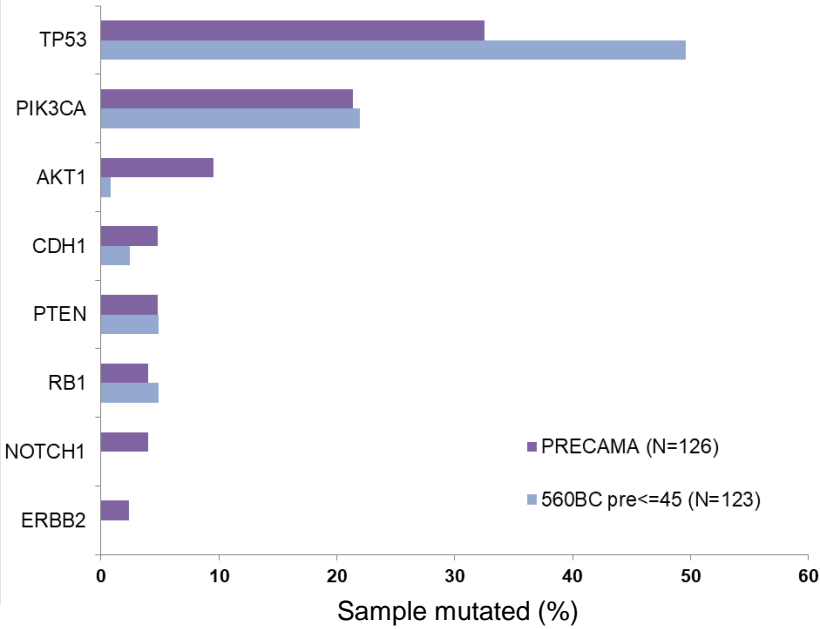

B

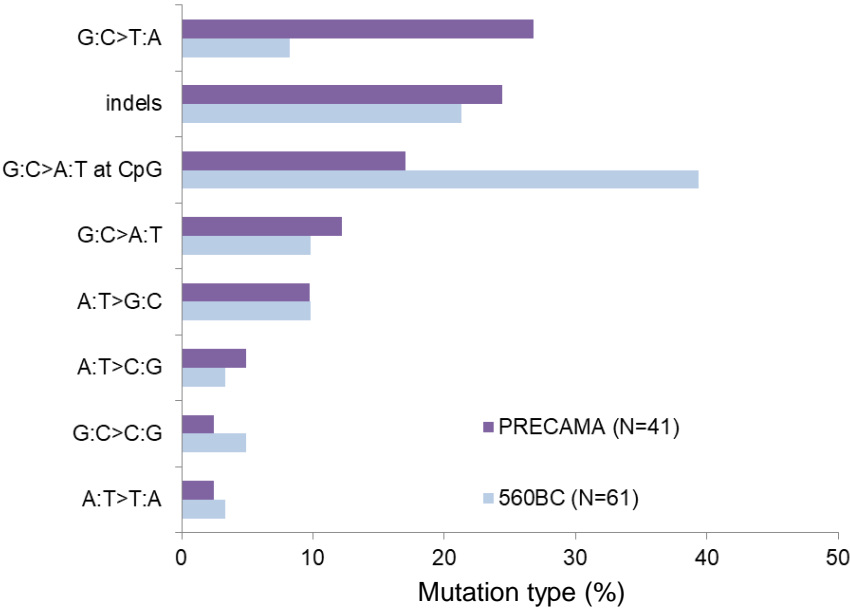

C

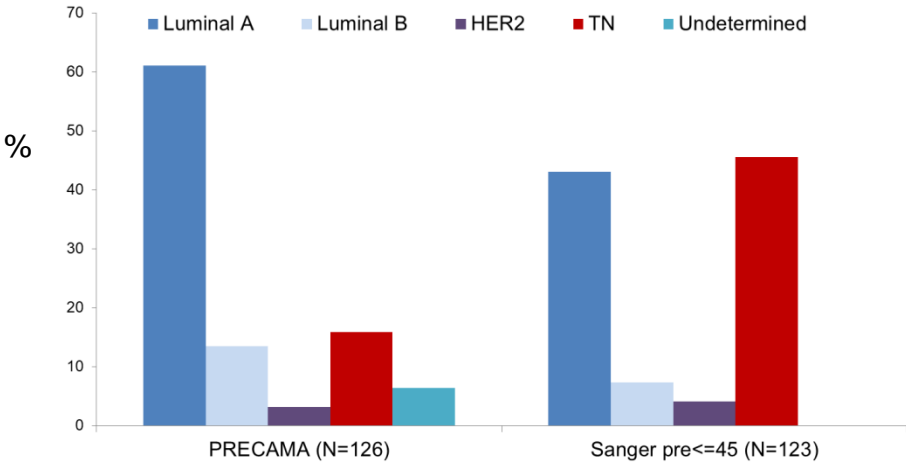

Supplement: S2 Fig — Data on 123 preBC cases with receptor status information reported in Nik-Zainal et al. (2016) were retrieved from supplementary materials (clinical information) or from COSMIC (mutation data) [6]. (A) Occurrences of mutations in the 8 BC genes analyzed in PRECAMA. (B) Distribution of TP53 mutation types in preBC cases. (C). Comparison of the distribution of IHC subtypes observed in preBC in the two datasets. Luminal A: ER+/HER2-; luminal B: ER+/HER2+; HER2-enriched: ER-/HER2+; triple-negative: ER-/PR-/HER2-. (PDF) [file pone.0210372.s003.pdf]
